# High-intensity exercise to promote accelerated improvements in cardiorespiratory fitness (HI-PACE): study protocol for a randomized controlled trial

**DOI:** 10.1186/s13063-019-3611-1

**Published:** 2019-08-08

**Authors:** Joshua E. McGee, Savanna G. Barefoot, Nicole R. Gniewek, Patricia M. Brophy, Angela Clark, Gabriel S. Dubis, Terence E. Ryan, Joseph A. Houmard, Paul Vos, Thomas D. Raedeke, Damon L. Swift

**Affiliations:** 1 Department of Kinesiology, East Carolina University, 388 Ward Sports Medicine Building, Greenville, NC 27858 USA; 2 Human Performance Laboratory, East Carolina University, 388 Ward Sports Medicine Building, Greenville, NC 27858 USA; 3 Department of Physiology, Brody School of Medicine, Greenville, NC 27858 USA; 4 The East Carolina Diabetes & Obesity Institute, East Carolina University, Greenville, NC 27858 USA; 5 Department of Biostatistics, East Carolina University, Greenville, NC 27858 USA; 6 Present affiliation: Department of Applied Physiology & Kinesiology, University of Florida, Gainesville, FL 32611 USA

**Keywords:** Methodology, Exercise intensity, African American, Cardiorespiratory fitness, Insulin sensitivity, Arterial stiffness

## Abstract

**Background:**

African Americans have a disproportionate prevalence and incidence of type 2 diabetes compared with Caucasians. Recent evidence indicates that low cardiorespiratory fitness (CRF) level, an independent risk factor for type 2 diabetes, is also more prevalent in African Americans than Caucasians. Numerous studies in Caucasian populations suggest that vigorous exercise intensity may promote greater improvements in CRF and other type 2 diabetes risk factors (e.g., reduction of glucose/insulin levels, pulse wave velocity, and body fat) than moderate intensity. However, current evidence comparing health benefits of different aerobic exercise intensities on type 2 diabetes risk factors in African Americans is negligible. This is clinically important as African Americans have a greater risk for type 2 diabetes and are less likely to meet public health recommendations for physical activity than Caucasians. The purpose of the HI-PACE (High-Intensity exercise to Promote Accelerated improvements in CardiorEspiratory fitness) study is to evaluate whether high-intensity aerobic exercise elicits greater improvements in CRF, insulin action, and arterial stiffness than moderate-intensity exercise in African Americans.

**Methods/Design:**

A randomized controlled trial will be performed on overweight and obese (body mass index of 25–45 kg/m^2^) African Americans (35–65 years) (*n* = 60). Participants will be randomly assigned to moderate-intensity (MOD-INT) or high-intensity (HIGH-INT) aerobic exercise training or a non-exercise control group (CON) for 24 weeks. Supervised exercise will be performed at a heart rate associated with 45–55% and 70–80% of VO_2_ max in the MOD-INT and HIGH-INT groups, respectively, for an exercise dose of 600 metabolic equivalents of task (MET)-minutes per week (consistent with public health recommendations). The primary outcome is change in CRF. Secondary outcomes include change in insulin sensitivity (measured via an intravenous glucose tolerance test), skeletal muscle mitochondrial oxidative capacity (via near-infrared spectroscopy), skeletal muscle measurements (i.e., citrate synthase, COX IV, GLUT-4, CPT-1, and PGC1-α), arterial stiffness (via carotid-femoral pulse wave velocity), body fat, C-reactive protein, and psychological outcomes (quality of life/exercise enjoyment).

**Discussion:**

The anticipated results of the HI-PACE study will provide vital information on the health effects of high-intensity exercise in African Americans. This study will advance health disparity research and has the potential to influence future public health guidelines for physical activity.

**Trial registration:**

ClinicalTrials.gov identifier: NCT02892331. Registered on September 8, 2016.

**Electronic supplementary material:**

The online version of this article (10.1186/s13063-019-3611-1) contains supplementary material, which is available to authorized users.

## Background

The American Diabetes Association (ADA) identifies racial health disparities in type 2 diabetes (T2D) as a major public health concern [1]. T2D prevalence in African Americans (AAs) is one of the greatest in the US; AAs have about 1.7-fold higher rates compared with their Caucasian American (CA) counterparts (females: 13.6% versus 7.4%; males: 14.1% versus 8.0%, respectively) [2]. An ADA position statement from 2016 [3] recognized the importance of physical activity to prevent T2D, as low levels are associated with greater T2D incidence. The prevalance of AAs meeting public health recommendations for physical activity is considerably lower than CA adults (56.5% versus 67.5%, respectively) [4]. Despite established racial disparities in T2D risk, AAs are under-represented in exercise research (i.e., sample sizes inadequate for sub-group analyses and few randomized clinical trials specifically examining AAs). This lack of data critically limits the federal Physical Activity Guidelines from making accurate conclusions on the effects of physical activity on health outcomes in AAs [5].

Low cardiorespiratory fitness (CRF) is an independent risk factor for T2D incidence [6–11]. However, an incremental dose response has been observed between CRF level and T2D incidence [8, 12]. Categorically defined “low CRF” is associated with the greatest risk of T2D, and a greater proportion of AAs have “low CRF” than CAs [8, 10, 13–27]. Higher intensities of aerobic exercise show promise in eliciting a larger magnitude of improvement in insulin sensitivity compared with moderate intensity. Moreover, arterial stiffness, another racial disparity identified in AAs, has shown a greater decline from high-intensity exercise than moderate intensity [28–30]. An additional contributor to the T2D racial disparities is the lower oxidative characteristics and subsequent reduced insulin sensitivity of skeletal muscle in AAs compared with CAs [13, 26, 31–33]. AAs tend to have a greater proportion of type II muscle fibers (i.e., less oxidative, vascularized, lower proportion of GLUT-4 transporters, and more insulin-resistant than type I fibers) compared with CAs [32, 34, 35]. A major adaptation to aerobic training is the shift in both type I and II fibers toward more oxidative properties (e.g., increased mitochondria size, density, and enzymes) and increased sensitivity to insulin (increased GLUT-4 expression) [36, 37]. Thus, lower CRF levels in AAs may contribute to the racial health disparities in T2D.

Data examining CAs suggest that high-intensity aerobic training results in greater improvements in CRF, insulin action, and arterial stiffness compared with moderate intensity [26, 38, 39]. Thus, high-intensity exercise may improve the low-CRF, stiffened-artery, and insulin-resistant disposition observed in obese AAs more readily because of greater shear rates in the vasculature, recruitment of type II fibers, and greater energy expenditure rates than moderate intensity. There are, however, no current randomized controlled trials comparing the health benefits of different exercise intensity training programs in AAs despite the greater T2D risk in AAs than CAs.

The goal of the HI-PACE (High-Intensity exercise to Promote Accelerated improvements in CardiorEspiratory fitness) study is to evaluate the effects of exercise intensity on CRF, insulin action, and arterial stiffness in AAs at high risk for T2D. The purpose of the following article is to describe the design, rationale, and methodology of the HI-PACE study.

### Specific objectives

The objective of the HI-PACE study is to determine whether high-intensity aerobic exercise training results in greater improvements in CRF, insulin action, arterial stiffness, mitochondrial function, adiposity, and quality of life compared with moderate intensity. Thus, the primary outcome of the HI-PACE study is the change in CRF following the intervention. Main secondary outcomes include change in insulin sensitivity (measured via an intravenous glucose tolerance test [IVGTT]), skeletal muscle mitochondrial oxidative capacity (via near-infrared spectroscopy [NIRS]), skeletal muscle measurements (i.e., citrate synthase, COX IV, GLUT-4, CPT-1, and PGC1-α), arterial stiffness (via carotid-femoral pulse wave velocity [PWV]), body fat, C-reactive protein, and psychological outcomes (quality of life/exercise enjoyment).

Participants will be randomly assigned to one of three groups: (1) moderate-intensity (MOD-INT), (2) high-intensity (HIGH-INT), or (3) a non-exercise control (CON) group for 24 weeks. The exercise volume for both exercise groups will be 600 metabolic equivalents of task (MET)-minutes per week (3–4 sessions per week), which is consistent with the current public health guidelines (500–1,000 MET-minutes) [5]. Participants in the MOD-INT group will exercise at the heart rate associated with 45–55% of maximal oxygen consumption (VO_2_ max), and participants in the HIGH-INT group will exercise at the heart rate associated with 70–80% VO_2_ max. Fitbit Flex accelerometers (Fitbit Inc., San Francisco, CA, USA) will be worn on the wrist by participants in all randomization groups to objectively monitor non-exercise physical activity during the intervention (devices will be removed during training sessions).

## Methods/Design

### Inclusion/exclusion criteria

The main inclusion and exclusion criteria for HI-PACE are shown in Table 1. The HI-PACE study is designed to intervene in sedentary, overweight, and obese AAs at high risk for T2D. Thus, we plan to enroll 60 sedentary, overweight, and obese AA adults (body mass index [BMI] of 25.0–45.0 kg/m^2^ and age of 35–65 years). All participants will be sedentary/low active and not participating in exercise training at the time of enrollment (<20 min and ≤2 days per week for the last 3 months). Major exclusion criteria for the HI-PACE study include diagnosed type 1 or 2 diabetes (or fasting glucose of more than 125 mg/dL or use of diabetes medication), known cardiovascular diseases (e.g., heart failure, serious arrhythmias, and peripheral vascular disease), previous stroke or myocardial infarction, excessively high resting systolic (>180 mm Hg) or diastolic (>100 mm Hg) blood pressure, significant medical conditions, life-threatening conditions, pregnancy or plans to become pregnant, and other medical conditions that are contraindicated for exercise training. Additionally, individuals who plan to diet, engage in weight loss, or demonstrate non-compliance during screening visits will be excluded.

**Table 1:** Major inclusion and exclusion criteria

Inclusion criteria	
Age	35–65 years
Sex	Men and women
Overweight/obese body mass index	25.0–45.0 kg/m^2^
Physically inactive	Sedentary/low active, not participating in regular aerobic or resistance exercise < 20 min, ≤2 days/week for last 3 months
African American	Self-identify as African American
Informed consent	Willingness and capability to provide written consent and to understand the exclusion criteria
Exclusion criteria	
Diabetes	Diagnosed type 1 or type 2 diabetes or fasting glucose ≥126 mg/dL
Cardiovascular disease or disorders	Diagnosed congestive heart failure, serious arrhythmias, peripheral vascular disease with intermittent claudication, previous stroke, or myocardial infarction
Resting blood pressure	Excessively high resting systolic (>180 mmHg) or diastolic (>100 mmHg) blood pressure. Participants taking blood pressure medications at time of recruitment are permitted to enroll.
Blood lipids	Total cholesterol ≥240 mg/dL, low-density lipoprotein cholesterol ≥160 mg/dL, or triglycerides ≥300 mg/dL
Other exclusionary medical conditions	Chronic or reoccurring neuromuscular, respiratory, gastrointestinal, neurological, HIV, or psychiatric conditions. Musculoskeletal conditions affecting exercise. Current treatment for mental illness or hospitalization from mental illness within previous 5 years. Autoimmune or collagen vascular diseases. Other medical conditions that are considered life-threatening or that can be provoked from exercise training
Other exclusion criteria	Pregnancy or plans to become pregnant. Currently engaging in or plans to engage in weight loss or dieting program. Addition of medication or dosage (or both) unstable in past 3 months. Previous bariatric surgery or current weight loss medications. Plans to leave the Pitt County (NC) area for more than 2 weeks during the next 6 months. Non-compliance in wearing pedometer or demonstration of high risk for non-compliance/dropout during screening

The study protocol has been approved by the East Carolina University (ECU) institutional review board and is registered on ClinicalTrials.gov (NCT02892331). This study protocol was prepared on the basis of the SPIRIT (Standard Protocol Items: Recommendations for Interventional Trials) guidelines. This SPIRIT Checklist is available as Additional file 1.

### Recruitment and pre-screening

A detailed summary of the study visits is described in Table 2. Recruitment material will be disseminated through newspaper (general readership and an AA-specific newspaper), targeted social media advertisement (e.g., Facebook and Instagram), email sent through company employee listservs (i.e., ECU, Pitt Community College, and Greenville government), and local organizational contacts in the Pitt County, North Carolina area (e.g., churches, physician offices, libraries, and barbershops). In addition, a study website will be created to provide basic study information and to serve as a mechanism for web-screening potential participants. Web-screening will be performed by using an online survey in which basic inclusion/exclusion criteria questions can be completed and subsequently reviewed by study staff. This online survey is created by using an online research database, REDCap (Nashville, TN, USA) [40], which is connected to the main study database. Interested individuals can also contact HI-PACE staff by calling the study phone number or by directly emailing the research coordinator. After this, study staff will phone-screen individuals for major aspects of the inclusion/exclusion criteria and provide additional information about study participation. Individuals who are eligible and still interested after phone screening will progress to screening visit 1.

**Table 2:** Detailed summary of data collection at study visits

Screening visit and informed consent	
- Informational session about study requirements	
- Obtain informed consent	
- Verify inclusion criteria (i.e., BMI and blood pressure)	
- Physical exam/review of medications	
- Non-exercise physical activity via activPAL	
- Exercise calendar and Barriers screening forms	
- Complete metabolic panel, lipids, insulin, C-reactive protein, and blood chemistries	
Baseline	
- PWV, muscle biopsy, IVGTT, and NIRS	
- SF-36 and FFQ	
- Body weight, blood pressure, anthropometry, DEXA, and maximal exercise test	
Randomization	
- CON, MOD-INT, or HIGH-INT group	
Mid-intervention (12 weeks)	
- Waist circumference	
- Body weight	
- Maximal exercise test	
Follow-up (24 weeks)	
- Non-exercise physical activity via activPAL	
- PWV, muscle biopsy, IVGTT, and NIRS	
- SF-36 and FFQ	
- Complete metabolic panel, lipids, insulin, C-reactive protein, and blood chemistries	
- Body weight, blood pressure, anthropometry, DEXA, and maximal exercise test	

*Abbreviations*: *BMI* body mass index, *CON* non-exercise control (group), *DEXA* dual-energy x-ray absorptiometry, *FFQ* food frequency questionnaire, *HIGH-INT* high-intensity exercise (group), *IVGTT* intravenous glucose tolerance test, *MOD-INT* moderate-intensity exercise (group), *NIRS* near-infrared spectroscopy, *PWV* pulse wave velocity, *SF-36* short-form health survey

### Screening visits

Screening visits will be conducted at the East Carolina Heart Institute by the research coordinator. During screening visit 1, the research coordinator will describe all properties of study participation, answer questions from individuals, and obtain informed consent. Following consent, the research coordinator will screen participants for the full inclusion/exclusion criteria, collect contact/demographic information, and review prescribed medications (individuals will be required to bring in prescribed medications for verification). For inclusion/exclusion purposes, height and weight (without shoes) will be measured to calculate BMI (in kilograms per square meters), and seated resting blood pressure will be assessed via an automated blood pressure monitor (HEM-907XL, Omron Healthcare Co., Ltd., Kyoto, Japan).

Individuals will be screened for ample time to participate in the exercise intervention by filling out an exercise calendar form, in which they will be asked to identify specific days and times (and back-up times) they are available to exercise at our facility (Additional file 2: Appendix A). Staff will also conduct a standardized interview with potential participants in which (a) weekly time commitments, (b) responsibilities for family care (i.e., child and elder), (c) distances of home and work from our exercise facility, (d) personal motivations for exercising, (e) levels of familial support, and (f) any other barrier(s) that would affect study adherence will be evaluated (Additional file 2: Appendix B). The exercise calendar and interview are intended to screen out individuals depicting high risk for non-compliance or dropout (or both) during the 24-week study. Previous studies using similar methodologies exhibited high exercise training adherence and study retention [41, 42].

Individuals who are still eligible at this point will wear a Fitbit Flex (Fitbit Inc.) and an activPAL accelerometer (PAL Technologies Ltd., Glasgow, UK) for 7 continuous days to assess baseline non-exercise physical activity level. The Fitbit Flex will be worn on the non-dominant wrist to obtain data on steps, miles, intensity, and calorie expenditure each day of wear (blinded to individual). Study staff will apply the activPAL by rolling a nitrile sleeve over the entire device and wrap an 8 × 10 cm sheet of transparent medical dressing completely around it to act as a waterproof barrier. Staff will rub an alcohol-based prep pad around the site, place the distal end of the activPAL toward the knee, and apply a separate sheet of medical dressing over the monitor to complete application to the leg (waterproofing method). The activPAL accelerometer will be worn on the individual’s mid-thigh and will not be removed for the entire 7 days. The activPAL measures postural aspects of time spent sitting/lying down, standing, and walking in hours per day as well as energy expenditure (MET-hours per day), steps per day, and number of sit-to-stand transitions.

The HI-PACE study will use a REDCap database to store all information collected from screening visits (e.g., contact/demographic information and blood lab results) and to track physical activity data during the physical activity assessment. During each day the devices are worn, REDCap surveys will be sent automatically to individuals’ email address to ask whether the activPAL and Fitbit devices were worn on the previous day and whether there were any extended periods of non-wear time. The purposes of the survey are to (1) increase the accuracy of the physical activity assessment by being able to eliminate days affected by non-wear and (2) prompt individuals to wear the devices consistently. Since changes in non-exercise physical activity can confound exercise-related changes in outcome measures [43, 44], it is necessary to ensure that participants in the HI-PACE study can regularly wear the devices.

Following completion of the baseline physical activity assessment (7 days), individuals will return in the fasted state to the East Carolina Heart Institute for screening visit 2 in the morning. The study nurse will perform a fasting blood draw and immediately send the sample to a clinical laboratory (LabCorp Inc., Burlington, NC, USA) for complete metabolic panel, lipid panel, insulin level, and blood chemistries. Pre-menopausal women will be required to complete a pregnancy test. The Fitbit and activPAL will be retrieved for accelerometer data to be downloaded and recorded in the study database. Upon completion of the screening visits, individuals will be scheduled for the baseline assessment visit.

### Assessment visits (baseline, mid-intervention, and follow-up)

**Fig. 1:**
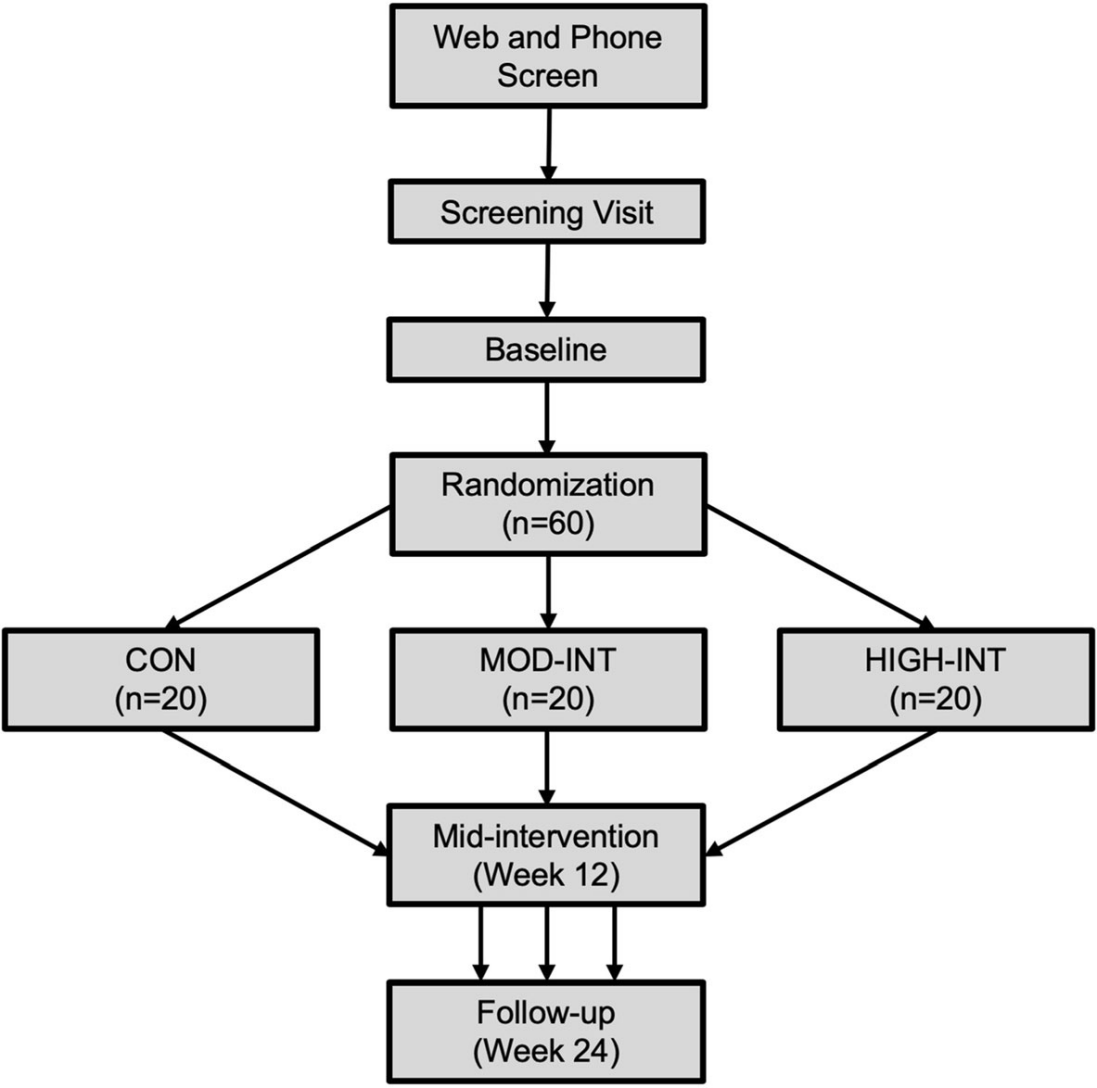
Flowchart of study visits in the HI-PACE (High-Intensity exercise to Promote Accelerated improvements in CardiorEspiratory fitness) study

**Fig. 2:**
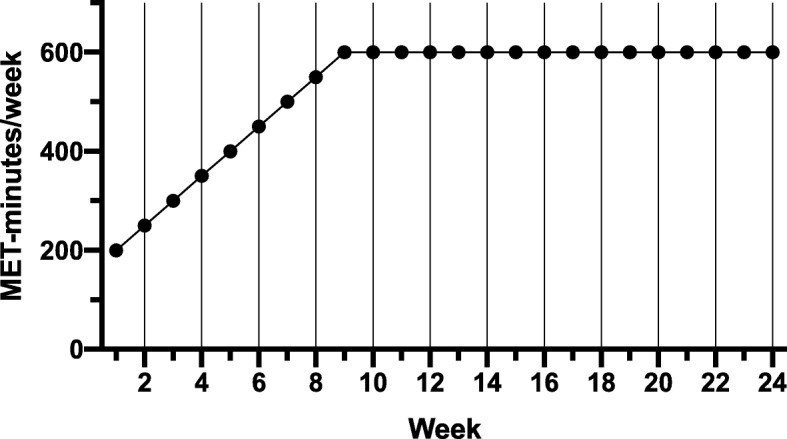
Study schedule of enrollment, intervention, and assessments. *Abbreviations*: *AIx* augmentation index, *CON* non-exercise control group, *DEXA* dual-energy x-ray absorptiometry, *FFQ* food frequency questionnaire, *HIGH-INT* high-intensity exercise group, *IVGTT* intravenous glucose tolerance test, *MOD-INT* moderate-intensity exercise group, *NIRS* near-infrared spectroscopy, *PA* physical activity, *PWV* pulse wave velocity, *SF-36* short-form health survey, *t*_*1*_ baseline, *t*_*2*_ mid-intervention (week 12), *t*_*3*_ follow-up (week 24)

A flowchart of the present study is shown in Fig. 1. Primary (i.e., CRF) and secondary outcome measures (i.e., arterial stiffness, mitochondrial measures, insulin sensitivity, skeletal muscle oxidative capacity, quality of life, and food-frequency questionnaires [FFQ]) will be obtained at baseline and follow-up (week 24). At mid-intervention (week 12), CRF, resting blood pressures, and anthropometry (i.e., body mass, waist circumference, and BMI) will be re-evaluated. Measurements of arterial stiffness, muscle biopsy, IVGTT, and NIRS will be obtained in this order during the same visit (baseline and follow-up), whereas CRF, anthropometry, and body composition will be obtained during the same visit of a separate week. The primary outcome will be obtained at the Human Performance Laboratory in the Ward Sports Medicine Building, whereas the secondary outcomes will be obtained at the East Carolina Heart Institute. Randomization into a study group will occur upon completion of all baseline assessments. For an overview of the schedule of enrollment, randomization, intervention, and assessments, see Fig. 2 for the completed SPIRIT figure.

### Primary outcome: change in maximal oxygen uptake (VO_2_ max)

The primary outcome measurement is CRF due to the well-established association between CRF levels and risk of T2D [8, 10, 11, 27]. We will measure CRF as VO_2_ max (the gold standard) from a maximal exertion treadmill test under the supervision of a physician. Maximal exercise testing will be conducted on a treadmill (Cardiac Science TM65, Davis Medical Electronics, Bothell, WA, USA) under a modified Balke protocol. For the warm-up, participants will initially walk at a speed of 2.0 mph at 0% grade for 2 min. Subsequently, we will increase treadmill speed to 3.0 mph to begin the treadmill test. During the test, we will increase the treadmill grade by 2.5% every 2 min until volitional exhaustion. Gas exchange (i.e., VO_2_ and carbon dioxide production (VCO_2_)) and pulmonary ventilation will be measured continuously by using a TrueOne 2400 Metabolic Measurement Cart (Parvo Medics, Salt Lake City, UT, USA). Heart rate, blood pressure, rating of perceived exertion, and electrocardiogram will be monitored and recorded before, during, and after the exercise test. The electrocardiogram will be cleared by the study physician prior to participant randomization. A valid maximal exercise test will meet two of three end criteria: (1) elevated respiratory exchange ratio (RER) (≥1.10), (2) plateauing of VO_2_, or (3) within ± 5 beats per minute of age-predicted maximal heart rate.

### Secondary outcome measures

Secondary outcome measures include change in insulin action, arterial stiffness, mitochondrial function, body fat, C-reactive protein, and psychological surveys.

### Insulin sensitivity

Insulin sensitivity will be assessed at baseline and follow-up (24 h following the last exercise session for the MOD-INT and HIGH-INT groups) via an IVGTT. After collection of fasting blood samples, glucose (dextrose 50%) will be injected into a catheter placed in the antecubital vein at a dose of 0.3 g/kg body weight. Subsequently, blood samples will be obtained at the following time points: 2, 3, 4, 5, 6, 8, 10, 12, 14, 16, 19, 22, 25, 30, 40, 50, 60, 70, 80, 90, 100, 120, 140, 160, and 180 min. Insulin will be injected at minute 20 of the test at a dose of 0.025 U/kg body weight. Blood samples will be centrifuged and stored at −80 °C until sample analysis for glucose and insulin. Insulin sensitivity index will be determined through a minimal model [45]. Follow-up IVGTT will be assessed within 18–24 h of the MOD-INT and HIGH-INT participants’ last exercise session.

### Arterial stiffness

Carotid-femoral PWV and aortic blood pressure parameters will be measured by using a SphygmoCor XCEL (AtCor Medical, Itasca, IL, USA). Carotid-femoral PWV, an index of the degree of arterial stiffness, is the gold-standard measurement of arterial stiffness [46]. Arterial stiffness measurements will occur during the morning in a quiet, temperature-controlled room at baseline and follow-up. Prior to each measure, participants will refrain from vigorous exercise, tobacco, caffeine, and alcohol for at least 12 h as well as large meals for at least 6 h. Participants will take their prescribed medications which will be logged and repeated at follow-up. The methodology for arterial stiffness measurements will adhere to a position stand by the American Heart Association [47].

For aortic blood pressure and stiffness measurements, participants will be in the seated position for a 5-min rest period. Following rest, aortic blood pressure (e.g., brachial blood pressures and aortic blood pressures) and stiffness (e.g., augmentation index and wave reflection) parameters will be obtained on the basis of acquisition of brachial artery pressure waveforms with the application of a generalized transfer function to derive the central aortic pressure waveform, from which estimates of aortic blood pressures are generated. Three measurements will be performed for aortic blood pressure parameters with a 1-min rest period between each measurement.

Subsequently, PWV will be obtained in the supine position following a 15-min rest. During rest, body surface measurements will be measured via a Gulick tape measure (Baseline, Fabrication Enterprises, White Plains, NY, USA) in triplicate to determine the distance traveled by the pulse wave between the carotid and femoral artery sites. Study staff will palpate and mark the carotid artery pulse (between the larynx and sternocleidomastoid muscle in the neck), the sternal notch (superficial landmark of aortic arch), and femoral artery pulse (over the ventral thigh halfway between the pubic symphysis and anterior superior iliac spine) [48]. The mean distance for each site will be used for PWV calculation. Pressure waveforms at the carotid arterial site will be acquired via applanation tonometry and electrocardiographic gating and the femoral arterial site will be simultaneously acquired by using the oscillometric device within the SphygmoCor XCEL. The PWV measurement will be conducted in duplicate, and the mean of these measurements will be the reported value. Both measurements must be within 0.5 m per second to be considered acceptable for data purposes. If the two measurements differ by more than 0.5 m per second, a third measurement will be obtained, and the reported value will be the median of the three measurements.

### Mitochondrial function

A percutaneous muscle biopsy (100–200 mg of tissue) will be obtained by using sterile techniques at baseline and follow-up from the vastus lateralis with a 5-mm Bergstr﻿öm muscle biopsy cannula with suction (Stille Surgical Instruments, Eskilstuna, Sweden), as previously described [49]. Briefly, participants will lie supine with legs extended (0° flexion) and two operators will spray ethyl chloride on the biopsy site and administer 1% lidocaine at each level of subcutaneous tissue, stopping superficial to the fascia. Following 2–3 min to allow for the local anesthetic effects, a 1-cm incision will be made through the skin and subcutaneous tissues, parallel to the femur, until an incision is made through the muscle fascia. The operator will use the biopsy cannula to locate the fascia incision site and advance the needle past the fascia, angled downward toward the floor to rapidly clip and collect the muscle sample with suction by the second operator. Following the biopsy, the sample will be trimmed of visible adipose tissue, weighed on a scale (AL54, Mettler-Toledo, Columbus, OH, USA), and snap-frozen in liquid nitrogen to be stored at −80 °C until analysis at study completion.

Peroxisome proliferator-activated receptor gamma coactivator 1-alpha (PGC1-α), COX IV, GLUT-4, and CPT-1 content will be determined; these proteins were selected as they are downstream of PGC1-α and represent distinct steps in oxidative metabolism [50]. About 50 mg of muscle tissue will be homogenized (T8 Ultra Turrax; IKA, Wilmington, NC, USA) in 20 volumes of cell lysis buffer (50 mM HEPES, 12 mM sodium pyrophosphate, 100 mM sodium fluoride, 100 mM ethylenediaminetetraacetic acid, 10 mM sodium orthovanate, 1% Triton X-100) supplemented with a protease and phosphatase inhibitor cocktails (Sigma-Aldrich, St. Louis, MO, USA). Lysates will be sonicated for 5 s, rotated for about 1 h at 4 °C, and centrifuged at 13,500 × g for 15 min at 4 °C. Protein concentration for each sample homogenate will be determined via a commercially available bicinchoninic acid protein assay kit (Pierce, Rockford, IL, USA). Aliquots containing 30 μg of total protein will be diluted in 4x Laemmli Buffer (Bio-Rad Laboratories, Inc., Hercules, CA, USA) with 5% β-mercaptoethanol (βME) at a 3:1 ratio prior to heating at 70 °C for 10 min. Denatured samples will be brought to room temperature, loaded onto a 10% polyacrylamide gel, separated by SDS-PAGE, and transferred to nitrocellulose membranes. Membranes will be blocked with Odyssey Blocking Buffer (OBB; Li-Cor, Lincoln, NE, USA) for 1 h and incubated with primary antibodies. Membranes will be washed with TBST (Tris-buffered saline, 0.1% Tween 20) and incubated with an anti-rabbit or anti-mouse fluorophore-conjugated secondary antibody (1:20,000; Li-Cor) in OBB supplemented with 0.1% Tween-20 for 1 h. Then the membranes will be washed with TBST followed by TBS prior to being scanned on the Odyssey CLx Imaging System (Li-Cor) and quantified on Image Studio software (V4.0.21; Li-Cor). GAPDH will be used as a loading control.

Citrate synthase activity will be determined with a colorimetric reaction by using reagents in a commercial kit (Sigma CD0720), as in a previous study [51]. A 10- to 15-mg piece of muscle will be diluted 20-fold in a buffer containing 100 mM KH_2_PO_4_ and 0.05% bovine serum albumin and homogenized at 4 °C by using the Ultra Turrax. Homogenates will undergo four freeze-thaw cycles before experimentation. Protein content will be measured by using the bicinchoninic acid assay, and citrate synthase activity will be assessed with reagents provided in the commercial kit (Sigma CS0720), which uses a colorimetric reaction to measure the reaction rate of acetyl coenzyme A and oxaloacetic acid.

### *In vivo* skeletal muscle mitochondrial oxidative capacity

As an additional measure of mitochondrial function, *in vivo* skeletal muscle mitochondrial oxidative capacity will be measured non-invasively via NIRS at baseline and follow-up. This NIRS approach measures the recovery kinetics of skeletal muscle oxygen consumption (mVO_2_) following brief exercise and has demonstrated strong correlations with current *in vivo* and *ex vivo* gold-standard measurements of mitochondrial function (i.e., magnetic resonance spectroscopy and muscle biopsy) [52, 53]. We will implement a NIRS testing protocol similar to that of Ryan et al. [53]. NIRS data will be obtained by using an OxiplexTS (ISS, Champaign, IL, USA), a frequency-domain tissue oximeter. Briefly, the OxiplexTS is equipped with two independent data acquisition channels and eight infrared diode lasers (four emitting at 691 nm and four at 830 nm) and a detector within each (emitter-detector distances of 2.0–4.0 cm). The absolute values of oxygenated hemoglobin (O_2_Hb) and deoxygenated hemoglobin (HHb) will be calculated in micromoles in accordance with the instructions of the manufacturer. Data will be collected at 4 Hz. Both NIRS probes will be calibrated prior to each test by using a phantom with known optical properties once the device warms up for at least 20 min.

For each NIRS measurement, participants will be supine on a padded table and have both legs extended (0° flexion). A skinfold caliper (Lange, Beta Technology, Santa Cruz, CA, USA) will be used to measure subcutaneous adipose tissue thickness at the probe site (about 10 cm above the patella). The NIRS probe will be secured to the skin at the vastus lateralis site with double-sided adhesive tape and Velcro straps. Additionally, a blood pressure cuff (Hokanson SC-10D or SC-10 L, D.E. Hokanson, Inc., Bellevue, WA, USA) will be placed proximal to the NIRS probe as high as anatomically possible to prevent unwanted signal noise from cuff inflation. A 15-gal air compressor (Model D55168, Dewalt, Baltimore, MD, USA) set to 30 psi will power a rapid-inflation system (Hokanson E20, D.E. Hokanson) to control the blood pressure cuff.

Upon securement of the probe and cuff, participants will complete a short-duration (about 10–30 s), submaximal, repeated knee extension or isometric quadricep exercise (or both) to increase mVO_2_. Following exercise, the recovery kinetics of mVO_2_ will be determined from a series of repeated arterial occlusions (275–300 mm Hg) for about 5–7 min in duration by using the following inflation/deflation timing: 5 s inflated/5 s deflated for about 90 s and then 10 s inflated/10 s deflated for the remainder of test. The beginning and end of each occlusion will be marked for calculations of mVO_2_ from the deoxygenated hemoglobin/myoglobin signal (i.e., slope during occlusion). The post-exercise mVO_2_ data will be fit to a mono-exponential function to calculate the rate constant, which is directly related to the mitochondrial respiratory capacity [52]. Three trials of exercise and occlusion procedures will be performed and the results will be averaged. Baseline and follow-up NIRS data will be analyzed via custom-written routines in MATLAB R2017b (MathWorks, Natick, MA, USA).

### Blood sample collection

A venous blood sample will be drawn with a 21-gauge needle with the participant in the fasted state at baseline and follow-up. A total of 21 mL of blood will be drawn by the study nurse and will immediately be sent to a clinical laboratory (LabCorp Inc.) for a complete metabolic panel, lipid panel, insulin level, and blood chemistries. Prior to glucose injection during the baseline and follow-up IVGTT, we will collect vials of archive plasma, serum, and red blood cells to be stored at −80 °C for future analysis. We also will send an additional serum separator tube to LabCorp Inc. for measurement of C-reactive protein.

### Anthropometry and body composition

Body weight will be measured in the fasted state via a calibrated scale (DigiTol 8510, Mettler-Toledo) (recorded to the nearest tenth of a kilogram). Dual energy x-ray absorptiometry (GE Lunar Prodigy Advance, Fairfield, CT, USA) will be used to measure body composition (fat and fat-free mass) at baseline and follow-up. Waist circumference will be measured via a Gulick tape measure at the natural waist (halfway point from the inferior border of the rib cage and the superior point of the iliac crest). Participants will be instructed to stand straight and upright with their feet together and arms to their side. Study staff will mark each landmark and measure the distance to determine the proper measurement site. For each measure, study staff will confirm that (1) the tape is parallel to the floor, (2) the tape touches the entire circumference of the participant, (3) the tape is not compressing any abdominal tissue, (4) the tape is not within abdominal folds, and (5) the measurement is recorded following a normal exhalation by the participant. Duplicate waist circumference measurements will be obtained. If measurements are ± 0.5 cm, the reported value will be the average of the two. If measurements differ more than 0.5 cm, a third measurement will be assessed, and the reported value will be the average of the three measurements. Waist circumference will be evaluated at baseline, mid-intervention, and follow-up.

### Non-exercise physical activity levels

Non-exercise physical activity data will be monitored in all randomization groups by using a Fitbit Flex activity tracker throughout the intervention period. Each group will be blinded to the number of steps accrued and instructed not to change their non-exercise physical activity levels from baseline. Prior to each exercise session, the Fitbit device will be removed from the participant (to not mix exercise and non-exercise physical activity data) and synced to the Fitbit software to upload their non-exercise physical activity levels. Participants in the CON group will sync their data at home by using the Fitbit software and be monitored by study staff to ensure compliance. Automated REDCap surveys will be emailed three times per week to all participants to inquire about Fitbit wear. Participants will be instructed to fill out these surveys to validate consistent device wear as this will allow staff to input non-exercise physical activity data on a weekly basis. This process helps to ensure consistent daily wearing of the device and to determine whether the participant did not wear the Fitbit for extended periods of time.

Study staff will use a database program (Fitabase, Small Steps Labs, San Diego, CA, USA) to centralize all non-exercise physical activity data (i.e., total daily steps, minutes of light, moderate and vigorous physical activity, miles traveled, and estimated kilocalorie energy expenditure). All non-exercise physical activity data synced to Fitabase will be stored in a custom-made REDCap database. Study staff will input all non-exercise physical activity data on a weekly basis.

### Dietary composition

Dietary intake will be tracked at baseline and follow-up via the Block FFQ [54]. The FFQ consists of 105 categorized items and assesses both frequency of consumption and portion size selections, in which participants will recall their typical eating habits within the previous 3 months at baseline and follow-up time points. The questionnaire estimates daily intake values of kilocalories and select macronutrients and micronutrients and also calculates servings by food group. The research coordinator will instruct participants at screening visit 1 to maintain current dietary habits and not to begin intentional dieting for the entirety of the study. Additionally, study staff will remind all participants on a weekly basis not to change their eating habits during the intervention. The FFQ serves as a semi-quantitative measure to ensure that dietary habits are not changed throughout the intervention.

### Psychological parameters

The short form health survey (SF-36) [55] will be used to measure quality of life at both baseline and follow-up. Exercise enjoyment will be assessed via the Physical Activity Enjoyment (PACE) Scale [56] and the Feeling Scale [57]. As secondary outcomes, the impact of exercise intensity on these affective responses (i.e., feelings of overall pleasure/displeasure and enjoyment) plays an important role in physical activity participation and adherence [58–60]. The PACE Scale is composed of eight items rated on a 7-point semantic differential scale in which “4” represents a neutral position. The PACE Scale will be collected every 4 weeks during the exercise intervention. Affective responses to exercise will be assessed by having participants complete the Feeling Scale. The Feeling Scale is a single-item, 11-point scale that assesses how individuals feel at a specific moment in time. The scale ranges from −5 (very bad) to +5 (very good), and 0 represents neutral feelings. Study staff will collect Feeling Scale data every 5 min during the first exercise session of every week of the exercise intervention.

### Randomization

Participants will be randomly assigned to the non-exercise control (CON), moderate-intensity (MOD-INT), or high-intensity (HIGH-INT) group upon completion of all baseline assessments and approval by the study physician. The study biostatistician will generate a randomization list to allocate participants in a 1:1:1 ratio to study groups. The randomization process will be performed by an individual separate from the research team, who has no interaction with the study participants or access to HI-PACE study data. All other research staff (including the principal investigator) will not have access to the randomization list. Once a participant has completed all baseline assessments, study staff will email the participant’s identification number and gender to the individual. The participant will be assigned to the next group on the randomization list. Upon randomization, the intervention period will begin the following week. A study flowchart is shown in Fig. 1.

### Aerobic exercise training

**Fig. 3:**
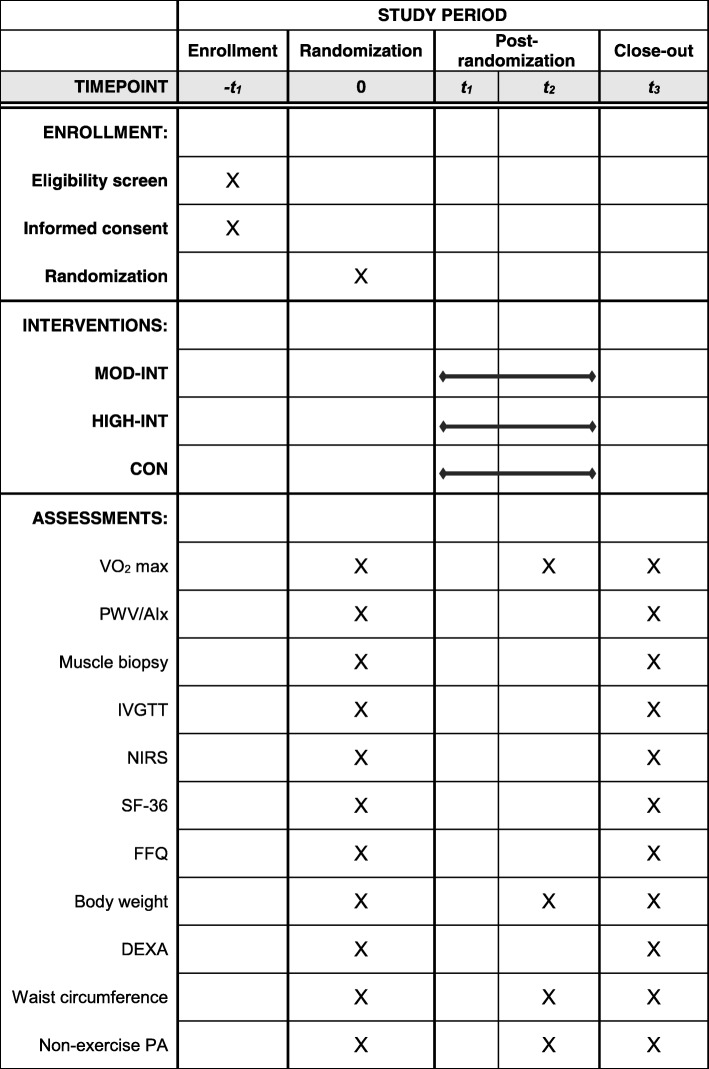
Ramping protocol of required MET-minutes in both the MOD-INT and HIGH-INT groups in the HI-PACE study. *Abbreviations*: *HIGH-INT* high-intensity exercise group, *HI-PACE* High-Intensity exercise to Promote Accelerated improvements in CardiorEspiratory fitness, *MET* Metabolic equivalents of task, *MOD-INT* moderate-intensity exercise group

All exercise sessions will be supervised by study staff and performed on a treadmill (Precor TRM 885, Precor Inc., Woodinville, WA, USA) to sustain control of energy expenditure from exercise. Participants in the MOD-INT group will exercise at a target heart rate associated with 45–55% VO_2_ max, and participants in the HIGH-INT group will exercise at a target heart rate associated with 70–80% VO_2_ max. The heart rate range for each participant will be determined on the basis of the maximal exercise test (baseline and mid-intervention). The full exercise dose for both groups will be 600 MET-minutes per week, which is consistent with current public health guidelines [5]. Since participants will be sedentary at baseline, we will increase the exercise dose incrementally throughout the study to avoid potential adverse events during exercise. Initially, the exercise dose will be 300 MET-minutes during week 1 and will increase by 50 MET-minutes per week until the maximum exercise volume of 600 MET-minutes is reached at week 9. The exercise dose will remain at 600 MET-minutes until conclusion of the intervention (Fig. 3). We will calculate the number of MET-minutes exercised on the basis of treadmill speed/grade and the participants’ weight by using the standard American College of Sports Medicine (ACSM) walking equation [61]. Custom-made Excel spreadsheets will be used to determine exercise time for each session on the basis of (1) the required weekly MET-minutes, (2) the participants’ weight, (3) exercise speed/grade, and (4) the amount of expected sessions per week (3–4 sessions per week).

At the first exercise session of each week, study staff will weigh participants (without shoes) on a calibrated scale and remind them not to alter their diet or engage in an exercise program outside of the study. Additionally, we will ask participants about any changes to their prescribed medications on a weekly basis.

Prior to starting exercise, participants will rest for 5 min in the seated position, after which study staff will measure systolic/diastolic blood pressures by using a mercury sphygmomanometer and record resting heart rate via a Zephyr Bioharness 3 monitor (Medtronic, Annapolis, MD, USA). Each participant will be instructed to complete a 5-min warm-up on the treadmill at a low speed (about 2.0 miles per hour) at 0% grade. Following completion of the warm-up, participants will begin their prescribed exercise by adjusting the treadmill’s speed or grade or both. During the supervised exercise, heart rate will be monitored continuously by using the Bioharness monitor to confirm exercise intensity and participants will be required to remain within their target heart range (MOD-INT: heart rate associated with 45–55% VO_2_ max; HIGH-INT: heart rate associated with 70–80% VO_2_ max). Heart rate, along with participants’ subjective rating of perceived exertion (RPE) via the Borg scale, will be recorded every 5 min [62]. The Feeling Scale will also be recorded every 5 min on the first exercise session of each week. Study staff will keep mobile laptop carts nearby the exercising participant(s) and will use custom-made Excel spreadsheets to quantify (1) the number of MET-minutes accumulated during exercise and number of MET-minutes remaining in the session and (2) mean heart rate and RPE of the session and (3) calculate the amount of time remaining in the current session. The spreadsheet calculates these variables in real time and can compensate for potential adjustments, such as increasing or decreasing treadmill speed or grade or both, during the exercise session.

Once completed with the exercise session, participants will perform a 5-min cool-down at a similar intensity as the warm-up. Subsequently, participants will rest for 5 min in the seated position for recording of post-exercise heart rate and systolic/diastolic blood pressures. Lastly, study staff will input all exercise session data (i.e., total exercise duration, MET-minutes, caloric expenditure, miles traveled, average speed, grade, heart rate, RPE, and percentage of time participant exercised within target heart rate range) into the database.

For each exercise session, the mean heart rate will be calculated by using Omnisense Analysis version 5.0 software (Medtronic). The Bioharness monitors continuously record heart rate data on a second-by-second basis. Thus, to collect the mean heart rate for each session, study staff will analyze heart rate data only during exercise by creating a time-specific sub-session annotation within the Omnisense software to exclude non-exercise heart rate data from calculation. This process will more accurately calculate exercise intensities during training sessions since heart rate will be measured continuously as opposed to intervals (e.g., every 5 min).

### Exercise economy

To address potential variability in exercise economy at a given workload, study staff will directly measure energy expenditure (EE) rate via indirect calorimetry (TrueOne 2400) at the participant’s prescribed exercise speed and grade on a treadmill [42, 63, 64]. This exercise economy test will be performed on weeks 1, 3, and 5 and then monthly until the conclusion of the intervention. The rate of EE determined through indirect calorimetry will be divided by estimated EE determined from the ACSM walking equation to develop a correction factor (i.e., actual EE rate/predicted EE rate). This correction factor will be used to (1) adjust the EE calculated from the ACSM equation to more accurately implement the exercise prescription which corresponds to 600 MET-minutes per week, (2) adjust participants’ exercise session time according to potential changes in metabolic or biomechanical efficiency (or both) from exercise training, and (3) verify required MET-minutes exercised by increasing or decreasing exercise session time accordingly.

### Training data management

Exercise volume adherence will be defined as MET-minutes exercised divided by required MET-minutes. Exercise intensity adherence will be quantified as time within the required target heart rate range divided by total exercise time. Exercise compliance will be defined as the number of sessions attended divided by the number of sessions required. The research team will actively monitor exercise volume/intensity adherence, compliance, and other indicators of intervention fidelity (e.g., target heart rate compliance, wear rate of accelerometer, participant morale, and progression rate of speed/grade) on a weekly basis in study meetings. In all randomization groups, Fitbit wear compliance will be monitored throughout the 6-month intervention. Weekly reports will be compiled from the study databases to monitor and review the compliance and adherence rates of all participants.

### Statistical considerations

The results of the current pilot study will be used to advise the design (effect size/statistical power) of a larger prospective intervention. The response variable for the primary outcome is change in VO_2_ max. The three treatment groups—CON, MOD-INT, and HIGH-INT—will be compared in terms of baseline VO_2_ max by using side-by-side boxplots and the corresponding numeric summaries along with mean and standard deviation. This will be repeated for post-treatment values of VO_2_ max and for VO_2_ max differences of post-treatment and baseline. If there are distributional concerns, log transformation of VO_2_ max will be considered. Unless there are extreme outliers or severe heteroscedasticity, one-way analysis of variance (ANOVA) will be used for inference regarding the primary outcome. The two-sample *t* test (without assuming equal variances) along with the associated confidence intervals will be used for differences in group means; confidence intervals for differences in group means obtained from the one-way ANOVA will also be reported.

The above steps used for VO_2_ max (the primary outcome) will be repeated for each of the numeric variables used for secondary outcomes. These variables are change in insulin sensitivity, mitochondrial protein content, citrate synthase activity, skeletal muscle mitochondrial oxidative capacity, arterial stiffness parameters, body fat percentage, and C-reactive protein. Data will be analyzed on an intention-to-treat basis.

The ordinal variables of exercise enjoyment and quality of life will be dichotomized into “no improvement” and “improvement”. Fisher’s exact test will be used to obtain an overall *P* value, and restriction to two of the treatment groups will provide estimated odds ratios and the associated confidence intervals.

The three groups will be compared by using the following demographic and other variables that may be related to exercise, change in VO_2_ max, or one of the other response variables: age, sex, body weight, BMI, waist circumference, body fat percentage, fat mass, fat-free mass, cholesterol, triglycerides, and blood pressures. If differences among the treatment groups in terms of one or more of these variables are deemed important, adjustments to the above comparisons will be made by using higher-order ANOVA, analysis of covariance (ANCOVA), or linear regression or a combination of these.

Power ranges from 0.85 for 15 participants per group to 0.96 for 20 participants per group using −0.112, 0.124, and 0.200 L/min as the means for the three groups on the basis of previous data [65], a common standard deviation of 0.250 L/min, and significance level α = 0.05. We expect attrition to be about 10–15% and so enrollment of 60 participants (20 per group) will be sufficient for the primary outcome.

All statistical analyses will be performed by using statistical software in R version 3.5.1. [66]. The resultant mean change and standard deviation of the change of outcome measures (if indicative of enhance cardiometabolic improvements in the HIGH-INT compared with the MOD-INT and CON groups) will be used for power calculations to determine the necessary sample size of a larger study.

## Discussion

The HI-PACE study has high public health relevance due to the increased disease burden of T2D and the lack of exercise training studies in AAs. HI-PACE will be the first study to compare two exercise training programs on multiple T2D and cardiovascular risk factors in overweight and obese AAs. HI-PACE has the potential to influence future physical activity recommendations and advance health disparity research. Additionally, valuable psychological parameters of quality of life and enjoyment of exercise will be obtained, which will help determine whether high-intensity aerobic exercise is a feasible strategy over a 6-month period to improve health outcomes in AAs. We anticipate that the results of the HI-PACE study will provide clear evidence of health benefits from high-intensity exercise, successful recruitment tactics, and favorable exercise adherence data in at-risk AAs. The pilot data will be necessary to conduct a larger-sample-sized exercise intensity study in AAs with adequately powered primary and secondary variables.

### Trial status

Participant recruitment for this study began in November 2016 and is ongoing. The study recruitment is expected to end in October 2019.

## Additional files

Additional file 1: SPIRIT (Standard Protocol Items: Recommendations for Interventional Trials) 2013 checklist. (DOC 122 kb)

Additional file 2: Appendices (Appendix A: Exercise calendar form; Appendix B: Barriers screening form). (DOCX 52 kb)

## Data Availability

Not applicable.

## Abbreviations

AA: African American
ACSM: American College of Sports Medicine
ADA: American Diabetes Association
ANOVA: Analysis of variance
BMI: Body mass index
CA: Caucasian American
CON: Non-exercise control (group)
CRF: Cardiorespiratory fitness
ECU: East Carolina University
EE: Energy expenditure
FFQ: Food frequency questionnaire
GLUT-4: Glucose transporter type 4
HIGH-INT: High-intensity exercise (group)
HI-PACE: High-Intensity exercise to Promote Accelerated improvements in CardiorEspiratory fitness
IVGTT: Intravenous glucose tolerance test
MET: Metabolic equivalents of task
MOD-INT: Moderate-intensity exercise (group)
mVO_2_: Skeletal muscle oxygen consumption
NIRS: Near-infrared spectroscopy
OBB: Odyssey Blocking Buffer
PACE: Physical activity enjoyment scale
PGC1-α: Peroxisome proliferator-activated receptor gamma coactivator 1-alpha
PWV: Pulse wave velocity
RPE: Rating of perceived exertion
SPIRIT: Standard Protocol Items: Recommendations for Interventional Trials
T2D: Type 2 diabetes
TBST: Tris-buffered saline, 0.1% Tween 20
VO_2_ max: Maximal oxygen consumption
