# Supplementary material for: High-intensity exercise to promote accelerated improvements in cardiorespiratory fitness (HI-PACE): study protocol for a randomized controlled trial
Source: Trials. 2019 Aug 8;20:484. doi: 10.1186/s13063-019-3611-1 (PMC6686537; doi:10.1186/s13063-019-3611-1)
Supplement: Supplementary file 2 — Appendices (Appendix A: Exercise calendar form; Appendix B: Barriers screening form). (DOCX 52 kb) [file 13063_2019_3611_MOESM2_ESM.docx]

Appendix A. Exercise calendar form.

**HI-PACE EXERCISE CALENDAR**

| **MONDAY** | **TUESDAY** | **WEDNESDAY** | **THURSDAY** | **FRIDAY** |
| --- | --- | --- | --- | --- |
| **Morning Session**  **6-10am** | **Morning Session**  **6-10am** | **Morning Session**  **6-10am** | **Morning Session**  **6-10am** | **Morning Session**  **6-10am** |
| Estimate Start Time | Estimate Start Time | Estimate Start Time | Estimate Start Time | Estimate Start Time |
|  |  |  |  |  |
| **Evening Session**  **4-7pm** | **Evening Session**  **4-7pm** | **Evening Session**  **4-7pm** | **Evening Session**  **4-7pm** | **Evening Session**  **4-7pm** |
| Estimate Start Time | Estimate Start Time | Estimate Start Time | Estimate Start Time | Estimate Start Time |
|  |  |  |  |  |
|  |  | **SATURDAY MORNING ONLY:**  **7-10am** |  |  |
|  |  | Estimate Start Time |  |  |
|  |  |  |  |  |

Appendix B. Barriers screening form

| **Barriers screen: The following screening is to determine whether major potential issues may exist with compliance of the participant if randomized to the exercise arm of the trial.** | |
| --- | --- |
| What was it about the study that interested you? | __________________________________ |
| Do you have any childcare commitments? What are they? | O Yes  O No |
| What are your current childcare commitments? | __________________________________ |
| Do you think your current childcare commitments could affect your participation in HI-PACE or regularly attending exercise sessions (3-4 times/week)? | O Yes  O No |
| Do you have any eldercare commitments? | O Yes  O No |
| What are your current eldercare commitments? | __________________________________ |
| Do you think your current eldercare commitments could affect your participation in HI-PACE or regularly attending exercise sessions (3-4 times/week)? | O Yes  O No |
| Are you currently enrolled in classes? | O Yes  O No |
| In which classes are you currently enrolled? | __________________________________ |
| Do you think your current class commitments could affect your participation in HI-PACE or regularly attending exercise sessions (3-4 times/week)? | O Yes  O No |
| Are you actively involved with a church? | O Yes  O No |
| What responsibilities do you currently hold at church? | __________________________________ |
| Do you think your current church commitments could affect your participation in HI-PACE or regularly attending exercise sessions (3-4 times/week)? | O Yes  O No |
| Do you have excessive work commitments? | O Yes  O No |
| What responsibilities do you have at work? | __________________________________ |
| How predictable is your work schedule? (e.g. unexpected travel, staying longer than expected at work, etc.) | O Not predictable at all  O Somewhat predictable  O Mostly predictable  O Very predictable |
| Please discuss the elements of your schedule that are unpredictable? | __________________________________ |
| Do you think your current work responsibilities could affect your participation in HI-PACE or regularly attending exercise sessions (3-4 times/week)? | O Yes  O No |
| Daily Commitments  Childcare Eldercare Student Church Work | |
| If selected for the exercise arm of the study, do you accept that sessions will be conducted on a treadmill only? | O Yes  O No |
| If selected for the exercise arm of the study, exercise sessions will be conducted 3-4 times/week at our fitness center. Is this a feasible time commitment? | O Yes  O No |
| If selected for the exercise arm of the study, exercise sessions generally last for 30-60 minutes. Is this length of time acceptable? | O Yes  O No |
| How far do you live from the exercise facility? | O 5-10 min. O 10-15 min. O 15-20 min.  O 20-25 min. O 25-30 min. O 30+ min. |
| What is the best time for you to exercise? | O 7am-10am O 1pm-3pm O 4pm-7pm |
| What are the best days for you to exercise?  Monday Tuesday Wednesday Thursday  Friday Saturday | |
| Backup time to exercise? | O 7am-10am O 1pm-3pm O 4pm-7pm |
| Backup days to exercise?  Monday Tuesday Wednesday Thursday  Friday Saturday | |
| How would your close family and friends feel about your participating in this 24-week study? How will your time commitment affect their life and your other commitments? | __________________________________ |
| Is this a good time for you to commit to a study? | O Yes  O No |
| Do you anticipate leaving town for more than 2 weeks during the study? | O Yes  O No |
| Are there any busy times at work or home that will be coming up during the study? | O Yes  O No |
| Do you understand that your assignment to the study groups is a completely random process? (66% exercise, 33% control) | O Yes  O No |
| If you are randomized to the control group, will you adhere to the requirements of this group during the study? | O Yes  O No |

| **Barriers screen: Staff questions** | |
| --- | --- |
| Does the participant have ≥2 time commitments? | O Yes  O No |
| Has there been any indication that the participant does not want to adhere to the exercise prescription in any way? | O Yes  O No |
| Does the participant have the availability to exercise during the given times? | O Yes  O No |
| Does the participant seem to be a viable candidate for the study? | O Yes  O No |
| Please identify this person’s level of risk of drop-out based on the barrier screen  (1- Very low risk of drop-out, 3- moderate risk, 5- high risk of drop-out) | O 1 O 2 O 3 O 4 O 5 |
| Does the participant need to be discussed further with the staff before determining inclusion into the study?  *All participants at moderate risk (3 or higher should be discussed with PI and research team) | O Yes  O No |
| Did participant have major indicators of poor adherence? | O Yes  O No  O Discuss with PI and investigative team |
| Has participant filled out time calendar? | O Yes  O No |
| Did the calendar indicate a lack of time to participate in HI-PACE? | O Yes  O No |
| What were the indications of poor adherence identified? | High time commitments   Childcare issues  Eldercare issues   Transportation issues  Lives far from ECU   Indications participant will quit if randomized to control   Rescheduled many visits prior to Screening visit |
| Please fully describe adherence concern | __________________________________ |
